# Deciphering Cardiac Biology and Disease by Single-Cell Transcriptomic Profiling

**DOI:** 10.3390/biom12040566

**Published:** 2022-04-12

**Authors:** Le Wang, Shengshou Hu, Bingying Zhou

**Affiliations:** State Key Laboratory of Cardiovascular Disease, Fuwai Hospital, National Center for Cardiovascular Diseases, Chinese Academy of Medical Sciences and Peking Union Medical College, Beijing 100037, China; wangle@fuwai.com

**Keywords:** single-cell RNA sequencing, heart development and disease, induced pluripotent stem cells (iPSC)

## Abstract

By detecting minute molecular changes in hundreds to millions of single cells, single-cell RNA sequencing allows for the comprehensive characterization of the diversity and dynamics of cells in the heart. Our understanding of the heart has been transformed through the recognition of cellular heterogeneity, the construction of regulatory networks, the building of lineage trajectories, and the mapping of intercellular crosstalk. In this review, we introduce cardiac progenitors and their transcriptional regulation during embryonic development, highlight cellular heterogeneity and cell subtype functions in cardiac health and disease, and discuss insights gained from the study of pluripotent stem-cell-derived cardiomyocytes.

## 1. Introduction

The mammalian heart is a unique organ, owing to its chambered structure, lack of renewal capacity [1], electrical conduction [2], hemodynamics [3], and never-ceasing contractions [4]. Beneath these properties lie the vast varieties of cells that act in concert to execute these well-orchestrated functions, and, when things go awry, lead to cardiac dysfunction [5,6]. Addressing the fundamental mechanisms of physiological and pathological phenomena thus requires accurate comprehension of the cellular diversity and their dynamics, a goal that can only be achieved at the systems level and at single-cell resolution [7,8,9].

**Figure 1:**
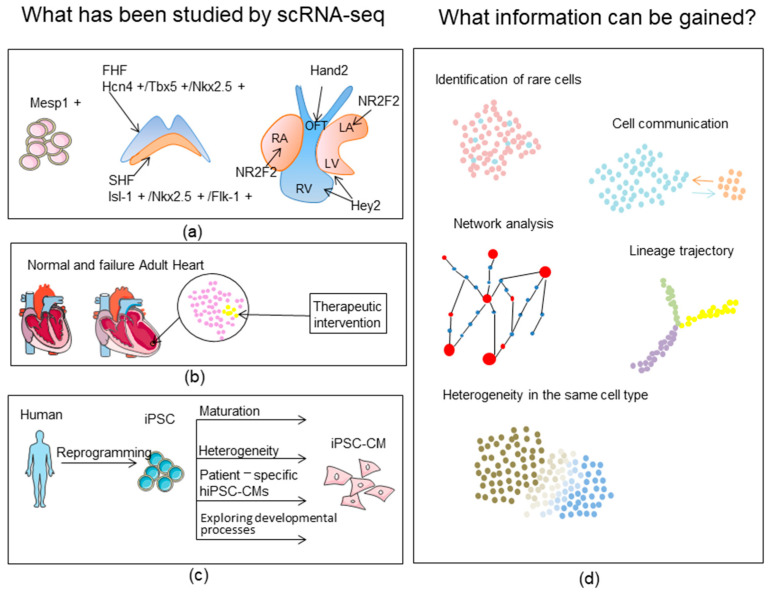
Topics commonly studied by scRNA-seq in the mammalian heart. (**a**) Embryonic heart development: the role of cardiac progenitor cells during in vivo cardiogenesis and critical transcription factors determining cardiac cell fates [12,14,17,20,21,22,23,24,25]. (**b**) Adult heart in health and disease: cellular heterogeneity and intercellular crosstalk, the role of specific cell subtypes in cardiac diseases, and potential therapeutic opportunities [8,9,13,15,16,26,27,28,29,30,31,32,33]. (**c**) Induced pluripotent stem-cell-derived cardiomyocytes: ways of improving modeling (maturation and reprogramming efficiency), patient-specific cells to understand genetic heart diseases, and in vitro modeling of embryonic heart development [18,34,35,36,37,38,39,40,41,42,43,44,45,46,47,48,49,50,51]. (**d**) ScRNA-seq can be used to explore cellular heterogeneity, identify rare cell types, deduce intercellular communications, establish regulatory networks, and construct lineage trajectories.

Single-cell RNA sequencing (scRNA-seq) is used to study the global transcriptomic profile of a single cell. Since its first implementation in 2009 [10], major advancements in scRNA-seq technology and its applications, along with the emergence of constantly expanding and diversified customized bioinformatic analysis tools, have substantially improved the accuracy, throughput, and versatility of large-scale scRNA-seq analyses [11]. Its most prominent applications include dissecting the cellular composition of organs, identifying rare cell populations, building trajectories of cellular dynamics, and mapping cell–cell interactions [8,12,13,14] (Figure 1d). Overall, scRNA-seq has transformed our understanding of the heart, particularly the mammalian heart [7,9]. The questions tackled by scRNA-seq include cellular heterogeneity of developing, mature, and diseased hearts; cardiac lineage commitment and its regulatory factors; cell subtype conversions and cell-crosstalk dynamics during cardiac disease progression; stem-cell reprogramming and differentiation, etc. [15,16,17,18]. From a translational viewpoint, scRNA-seq is set to open up a new field of single-cell-level diagnosis and cell-subtype-specific intervention of cardiovascular diseases [19].

In this review, we summarize what we have learned from scRNA-seq studies in the mammalian heart, including mouse and human, to enhance our perception of the cellular and molecular processes underlying cardiac development, homeostasis, and abnormalities. We also outline our knowledge gained from single-cell profiling studies that use stem-cell-derived cardiomyocytes, particularly with respect to the heterogeneity and trajectories of cell fates during cardiac differentiation, and how gene expression is regulated during somatic differentiation or somatic reprogramming, such that these findings may inform us of strategies to improve the cell model and of the pathophysiology of cardiac diseases (Figure 1). Finally, we provide our perspectives on future challenges and prospects of scRNA-seq and related techniques in further pushing the boundaries of our knowledge of the heart.

## 2. Single-Cell Transcriptomic Characterization of Embryonic Heart Development

### 2.1. ScRNA-seq Analysis of Cardiac Progenitor Differentiation

The heart is the first functional organ formed during mammalian embryonic development [52]. It is derived from the mesodermal (middle) germ layer that forms during the very early stages of embryonic development. On the cellular level, cardiogenesis can be defined as the process of mesodermal progenitor cells evolving into cardiomyocytes (CMs), endothelial cells, smooth muscle cells, cardiac fibroblasts, and other common cell types in the heart. The earliest cardiac progenitor cells (CPCs) originate in the anterior splanchnic mesoderm (often referred as the first heart field, FHF) and are responsible for the formation of the left ventricle and parts of the atrial chambers. Another anatomically and functionally distinct source of CPCs, termed the second heart field (SHF), originating from the splanchnic pharyngeal mesoderm, differentiates later than cells from the FHFs, and mainly gives rise to the right ventricle and the outflow tract. Proper heart formation requires the coordinated development of these two pools of progenitor cells. During cardiogenesis, FHF CPCs form the early cardiac tube, whereas cells of the SHF subsequently enter the developing tube and comprise the second lineage contributing to formation of the heart [53,54,55].

Transcriptional regulation of CPC formation, patterning, specification and lineage commitment has been characterized in great depth, and Mesoderm posterior 1 (*Mesp1*), a transcription factor of the basic helix–loop–helix (bHLH) family, marks the earliest CPCs and promotes their specification, epithelial–mesenchymal transition (EMT), and cardiovascular differentiation. Although only transiently expressed at the onset of gastrulation, it is known as a master regulator of multipotent CPC specification [56]. Hand2, another member of the bHLH family, is highly expressed in the early CPCs of the FHF and SHF, and declines thereafter [57]. Its function was found to be essential to the survival of SHF CPCs [58]. *Nkx2.5* is expressed in both heart fields. During development, its expression becomes shut down in the FHF to allow for cardiac differentiation. By contrast, CPCs in the SHF (*Nkx2.5^+^ Isl1^+^*) are maintained as a proliferating, non-differentiated population. Due to the constant cell-fate changes and migration of CPCs during development, previous studies mostly relied on lineage tracing experiments to track such sophisticated changes. While immensely useful at identifying cellular origin and fates, these techniques were not capable of distinguishing individual descendant cells of the same origin. Furthermore, the definition of cell states and lineages were based on a restricted set of known markers, impeding the discovery of new cell subpopulations.

ScRNA-seq offers an unbiased approach of interrogating the transcriptome of every single cell in a population. Therefore, it is particularly suitable for analyzing cellular heterogeneity, uncovering new cellular subtypes or states, and characterizing the molecular signatures of individual subpopulations. Another advantage is that bioinformatic algorithms allow for the deduction of molecular events between two sampling time points, providing insight into the continuum of alterations taking place between two static snapshots. Therefore, scRNA-seq has greatly advanced our knowledge of the cellular composition, cell fates, and their transcriptional, or even epigenetic, control during embryonic cardiac development (Table 1).

#### 2.1.1. *Mesp1*

Almost all cardiac cells are derived from *Mesp1*-expressing cells, including multipotent cardiovascular progenitors (MCPs) of both heart fields. Inactivation of *Mesp1* in mice resulted in severe cardiac malformations called ‘cardia bifida’, leading to embryonic lethality around E10.5. In *Mesp1*-null embryos, cardiac bifida has been attributed to a defect of cardiac mesoderm migration and is likely caused by failure of ventral fusion of the cardiac mesoderm [62,63]. The importance of this gene has therefore attracted many researchers into investigating its biological [64,65,66], as well as extra-physiological, roles [67,68]. However, evidence of cellular heterogeneity within a single-gene-labeled cell population emerged, which hinted at much more complex mechanisms underlying CPC lineage settlement and differentiation. For example, left ventricular progenitors express *Mesp1* earlier than atrial progenitors. Therefore, it had become necessary to dissect CPC biology in greater detail than before.

The heterogeneity of *Mesp1*-expressing cells at single-cell resolution was first described in a doxycycline-inducible *Mesp1* mouse embryonic stem cell line [59]. Forty-eight *Mesp^+^* mesoderm cells (day 4 of induction) were selected for scRNA-seq. A total of six subpopulations were identified by using hierarchical clustering. One of the clusters displayed little expression of lineage-regulatory factors and may represent a population of less differentiated cells, or may have committed to other mesoderm lineages. Three of the clusters displayed pro-cardiogenic expression signatures. One of them was characterized by concurrent *Isl1*, *Mef2c*, *Tbx5*, and *Myocd* expression. Another group demonstrated enrichment in *Isl1* only, whereas the third subcluster highly expressed *Hand2*, *Meis1,* and *Gata4*. These distinctions could reflect different cell fates, such as cells fated toward the FHF versus the SHF. The two final clusters comprised hematopoietic-primed cells, showing abundant *Etv2* expression. However, to what extent this in vitro system mimics in vivo development was uncertain.

Single-cell transcriptome profiling of 85 *Mesp1*-null mouse CPCs at E6.75 (prior to the appearance of developmental abnormalities) showed that cells were locked at the epiblast stage, evidenced by the upregulation of pluripotency genes (e.g., *Nanog*, *Eras*, and *Oct4*) and epiblast markers (e.g., *Cdh1*, *Epcam*, *Cldn6*, and *Cldn7*), as well as the downregulation of epithelial–mesenchymal transition- and cardiovascular-commitment-related genes, corroborating the role of *Mesp1* in controlling the exit of pluripotency [20]. Inspection of wild-type *Mesp1^+^* cells over time uncovered five distinct cellular fates of these cells. One group of cells was committed to an endothelial cell lineage, while another subset of cells exhibited CM differentiation. Two other subpopulations were anterior and posterior SHF progenitors, respectively, whereas the fifth population expressed endodermal markers. Both the in vitro model system [20] and the in vivo characterization identified pro-cardiac cell subsets, subsets of other mesodermal lineages, as well as a possibly non-mesodermal population. These studies provided evidence of the heterogeneity and multi-directional fates of *Mesp1*-expressing progenitor cells.

Spatially resolved single-cell transcriptomics of microdissected anterior cardiac regions of mouse embryos revealed a group of *Hand1*- and *Snail1*-positive, but *Nkx2.5*-negative, progenitor cells, that was termed the juxta-cardiac field (JCF), and the cells were shown to be progenitors of the FHF. A *Mesp1*-Cre driver line was used to show that it arose from *Mesp1*-positive mesoderm. Further analyses showed that the JCF contributed to CMs and the epicardium. Specifically, loss of function of the JCF caused reduction of the myocardium and the original epicardium, leading to early embryonic death [21]. The discovery of this pool of progenitors provided an important link between *Mesp1^+^* progenitors and early differentiated CMs.

#### 2.1.2. *Isl1*

The SHF develops from multipotent cardiovascular progenitors characterized by the expression of the transcription factor Isl1 [69]. Isl1 protein has been detected already at the cardiac crescent stage, suggesting the possibility that *Isl1* is expressed in the FHF at a very early stage [70]. Genetic fate-mapping has uncovered that multipotent *Isl1^+^* cardiovascular progenitors give rise to a subset of CMs pacemaker, endothelial, and smooth-muscle cells in vivo [71]. *Isl1^+^* CPCs are responsible for producing the majority of cells (~40%) in the developing heart [72], and a population of *Isl1^+^* cells was found to persist throughout life [72].

As with all other single-gene-labeled cell populations, *Isl1^+^* cells are also unlikely to represent a homogeneous and temporally stable cell set [22]. Indeed, scRNA-seq revealed five subpopulations of *Isl1^+^* cells in the developing mouse heart. Some of the clusters expressed cardiac transcription factors and sarcomeric genes, while others expressed *Cd31* and were predicted to be involved in endothelial cell differentiation. Low dimensional projection of these cells did not show strong stage-dependent clustering, and *Isl1* displayed decreased expression in differentiating cells, indicating its implication in the maintenance of CPC multipotency. Reconstruction of developmental trajectories of *Isl1^+^* CPCs corroborated their bifurcation into CM and endothelial cell expression programs. *Isl1*-knockout cells confirmed the necessity for *Isl1* in CPC fate bifurcation, because they were locked in a transition state that precedes bifurcation [22]. Since this study covered E7.5 to E9.5, and only showed bifurcation, it remains to be determined when *Isl1^+^* cells commit to other lineages, such as smooth-muscle cells and pacemaker cells, and whether those lineage segregations are *Isl1*-dependent.

#### 2.1.3. *Nkx2.5*

Transcription factor *Nkx2.5* is expressed upon cardiac crescent formation and exists in both FHF- and SHF-progenitors. *Nkx2.5* is a target of major regulators of embryonic development, such as *Mesp1* [56] and *Notch1* [73]. It is one of the best known regulators of cardiac lineage commitment, and, therefore, *Nkx2.5^+^* CPCs are indispensable for the development of CMs [74]. In vitro experiments have repeatedly demonstrated that the ectopic expression of *Nkx2.5*, *Gata4*, and *Tbx5* in neonatal murine cardiac fibroblasts is sufficient for their conversion into CMs [75,76]. During in vivo development, the expression of *Nkx2.5* is dynamic, being abundantly expressed in CPCs, temporally suppressed during CM differentiation, and then stably expressed at low levels thereafter [77]. *Nkx2.5* expression also varies by cell type [78], and early studies have explicitly shown that *Nkx2.5^+^* CPCs are multipotent [79]. These observations hinted at a highly heterogeneous and dynamic *Nkx2.5*-expression cell population, which could benefit from dissection at the single-cell level.

Early scRNA-seq studies concerning the developing mouse heart have validated the role of *Nkx25* in CM differentiation and maturation. Single-cell transcriptomic profiles of E9.5 *Nkx2.5*^−/−^ embryonic cells resembled wild-type CMs from the left atrium, and not the left ventricle, suggesting impairment in the transcriptional program of ventricular CM differentiation [12]. ScRNA-seq was also applied to an *Nkx2.5* haploinsufficiency model to characterize the effect of this gene on cardiac cell maturation. *Nkx2.5* seemed to exert little effect postnatally, since P0 and P21 cardiomcyocytes exhibited comparable maturation profiles. However, *Nkx2.5*^+/−^ cells at embryonic day 14.5 (E14.5) were clearly less mature than the wild-type control, characterized by the significantly lower levels of several maturation genes (e.g., *Ttn* and *Myh6*). Interestingly, the maturation of endothelial cells was also delayed. These findings highlighted the central role of *Nkx2.5* in mammalian CM differentiation and maturation.

Transcriptomic profiling of single FACS-purified *Nkx2.5* and *Isl1^+^* cells from three stages of the mouse embryo revealed three distinct *Nkx2.5* subpopulations, corresponding to early, intermediate, and late CPCs [22]. Although they exhibited many differentially expressed genes, all three subclusters of *Nkx2.5^+^* CPCs showed strong enrichment in gene ontology terms related to muscle development and contraction. A trajectory analysis of *Nkx2.5^+^* cells demonstrated that they were exclusively fated for CMs, supporting the common perception that *Nkx2.5* is associated with a myogenic cell fate. Specifically, it was shown experimentally that the continued expression of *Nkx2.5* was both required and sufficient to induce a stable CM fate by opening chromatin regions of CM-specific genes.

A similar scRNA-seq design was used by Xiong et al. [23] to elucidate the interplay between FHF and SHF CPCs. Macrophage migration inhibitory factor-C-X-C motif chemokine receptor 2 (*Mif-CXCR2*) chemotaxis was found to be a primary signal for SHF CPC migration to the forming heart tube. Notably, *Nkx2.5* was shown to directly bind to the enhancer regions of *Cxcr2/4*, promoting their specific expression in the SHF. Therefore, this study established an important role of *Nkx2.5* in the regulation of heart tube elongation by facilitating the expression of a chemokine receptor in SHF CPCs.

#### 2.1.4. *Hand2*

Expression of the mouse *Hand2* gene was first observed in maternally derived decidua at E7.5. Its expression in the lateral mesoderm in mouse embryos starts at E7.75. *Hand2*-expressing cells migrate toward the anterior most end of the embryo and form a horseshoe-shaped cardiac crescent. This *Hand2* expression continues until the linear heart tube stage at E8.5. Until cardiac looping begins, *Hand2* expression is restricted to the right ventricle and outflow tract. *Hand2* forms a heterodimer with *Twist1*. *Twist1* is a key regulator to maintain an appropriate level of the *Hand2* activity by antagonizing the function of *Hand2* in the developing limb bud. Accumulating data have provided evidence that balanced expression of *Hand2* and *Twist1* is essential for proper limb development, and disruption of the expression balance between these two genes causes developmental defects [80]. However, whether *Hand2* also mediates cardiac malformation and which subsets of cells mediate the effect *Hand2* on cardiac development are unknown.

De Soysa and colleagues [17] performed scRNA-seq on more than 36,000 cells from the cardiogenic region of the mouse embryo to understand transcriptional regulation of CPC specification. Using a Boolean-network-based lineage-specifier prediction method, they identified *Hand2*, *Tead2*, and *Arid3b* as cell fate determinants of the outflow tract. The identification of *Hand2* as a specifier for OFT, but not RV, was unexpected, due to its reported biological functions in the RV. A pseudotime trajectory analysis separated cells into three distinct states: an OFT state, which was almost devoid of *Hand2*-null cells; an RV1 state with comparable numbers of wild-type and mutant cells; and another RV state, RV2, which mostly comprised wild-type cells. This demonstrated that the loss of *Hand2* specifically abolished specification of OFT cells, but impaired RV differentiation to a lesser extent. It clearly would have been extremely difficult to decipher the cellular underpinnings of *Hand2*-regulated CPC specification without single-cell techniques.

### 2.2. Cellular Heterogeneity in Cardiac Development

While CPCs have been well characterized by using scRNA-seq, less is known about other cell types at relatively later stages of development. Specifically, there is particular interest as to what cell types constitute the developing heart, how they change with time, and whether they are a transcriptionally homogeneous or heterogeneous population.

In the early reports of the developing mouse heart, three major cell types were recovered from scRNA-seq data, including CMs [25], endothelial cells (EC), and fibroblast (FB)-like cells. At E9.5, FB-like cells were absent in the atria, ventricles, and OFT, but their proportion gradually increased to 30–45% of all ventricular cells at birth, accompanied by the reduction in the percentage of CMs. Although changing in number, their transcriptional profiles stayed unchanged during development. By contrast, the percentage of developing ECs remained relatively stable (10–15%), but showed temporal progression of gene expression. Atrial and ventricular CMs represent two distinct subtypes of CMs and could be distinguished by marker gene expression. Ventricular CMs were further distinguished by their anatomical location, i.e., left versus right ventricle, during the early stages of development (before E14.5), but this was not observed for later time points or non-CMs. From a different perspective, ventricular CMs could be subdivided into a subgroup with proliferative capacity, and another subgroup with FB-like expression. Thus, there is apparent spatial and temporal cellular heterogeneity in the developing mouse heart.

Ultimately, we are interested in how much of the knowledge acquired from animal models can be directly applied to humans. A scRNA-seq analysis of 3842 cardiac cells from human embryos (5 to 25 weeks of gestation) revealed nine clusters, which represented four major cells types: CMs, fibroblast-like cells, endothelial cells (ECs), and valvar cells [14]. The first three were very similar to the mouse study [25], while valvar cells were not identified in mice, possibly due to the low number of total cells acquired. Alternatively, this discrepancy could arise from the different sampling time points, because valvar cells were mainly from later-stage samples (22–25 weeks). Similar to mice, the proportion of CMs drastically decreased during human heart development, while the FB-like population markedly increased.

Human embryonic CMs consisted of compact and trabecular CM subtypes, each of which could be further classified into atrial and ventricular CMs. The distinction between atrial (high expression of transcription factors *NR2F1*, *FOS*, *HEY1*, *EGR2*, *CREB3L2*, and *HAND2*) and ventricular CMs (high expression of transcription factors *HAND1*, *HEY2*, *IRX3*, and *NFIA*) emerged as early as 5 weeks post gestation [25]. In addition, atrial and ventricular CMs were different between the left and right side of the heart. For example, *IRX3* and *HAND1* were specifically expressed in left-ventricular CMs, whereas *PITX2* expression was restricted to left-atrial CMs [7]. These lines of evidence indicated substantial heterogeneity of and strong spatial influence on CM phenotype and gene expression. Human cardiac fibroblasts were composed of two largely stage-dependent subpopulations: an early proliferative subgroup and a later subgroup that was more involved in extracellular matrix (ECM) organization [30]. By contrast, human cardiac ECs comprised four distinct subpopulations that reflected their anatomical origins: endocardial cells, coronary vascular ECs, vascular ECs, and valvar ECs [81]. It is apparent that both the developmental time and anatomical region determine the cellular phenotype, generating the observed cellular heterogeneity.

To better resolve the spatial aspect of human cardiac development, spatial transcriptomics was applied to a temporally more confined set of four human embryos (ranging from 4.5 to 9 weeks post-conception) [7]. Three types of CMs were identified, including atrial, ventricular, and *MYOZ2*- and *FABP3*-expressing CMs that were not restricted to a specific anatomical region. A *Myoz2*-expressing CMs subtype was also described in the adult mouse heart [29]. *FABP3*, a gene for fatty acid transport, was found to be highly expressed in more mature ventricular CMs in mice [25] and in humans [14]. FB-like cells were present in a conglomerate cluster that also contained epicardium-derived cells (EPDCs). FB-like cells were subdivided into a cluster at the base of the OFT and in heart valves, and another within the OFT that is potentially involved in its morphogenesis. The two EC populations corresponded to compact and trabecular myocardium, respectively. These findings support the notion that localization and function are key determinants of cardiac cellular subtypes. While the spatial analysis was tremendously useful at determining the precise locations of cells, a major limitation of this study was the lack of biological replicates, and that the four embryos were of different genders, which may confound data interpretation. Nevertheless, the findings in this study were largely concordant with previous studies in mice and humans, in that heterogeneity exists for all cardiac cell types, which is often related to time, location, and cellular function. This location-dependent effect is particularly prominent for CMs, which consistently exhibit chamber-specificity.

In comparison to myocardial cells, the cellular components, heterogeneity, and subtype markers in the cardiac conduction system remained elusive. An scRNA-seq of cells from three microdissected regions (sinoatrial node (SAN), atrioventricular node (AVN), and Purkinje fiber (PF)) established *Hcn4*, *Isl1*, *Shox2*, and *Tbx3* as SAN markers, while confirming one-fourth of the previously reported markers of the SAN identified by bulk RNA sequencing methods as genes more abundantly expressed in other cell types. Several novel SAN genes were uncovered, including *Igfbp5*, *Cpne5*, *Rgs6*, *Ntm*, and *Smoc2*. In the same vein, *Cpne5* was identified as a novel general AVN marker. AVN cells were further subdivided into six subclusters, several of which had been described in previous studies. Among the PF cells was a group of standard PFs, as well as transitional PF cells, marked by the expression of concurrent expression of *Ntm* and *Cpne5*. Importantly, the identification of clinically relevant cellular subtypes may inform us of disease pathology and hint at therapeutic opportunities [24].

## 3. Cell Heterogeneity and Cell Crosstalk in Adult Heart and Cardiovascular Diseases

Although cardiovascular diseases include a wide range of conditions and exhibit diverse phenotypes, they can all be traced back to pathological changes in cellular compositions, cell crosstalk, and molecular alterations. In particular, unique phenotypic changes of specific cell subtypes, embodied by dynamic alterations in the cell’s transcriptome, have been identified as key factors underlying pathological conditions, such as heart failure and cardiomyopathy [82]. ScRNA-seq allows for the unbiased identification of cell subpopulations and the characterization of significant phenotypic heterogeneity in an ostensibly homogeneous cell type. By analyzing the phenotypic characteristics and dynamics of CMs, fibroblasts, endothelial cells, macrophages, and other non-CMs in the cardiac network, we are able to uncover specific cell types and inter-cell subtype crosstalk that maintain homeostasis or contribute to disease progression. The identification of different subpopulations and rare cell subpopulations may also lead to their exploitation as targets of cardiovascular diseases (Table 2).

### 3.1. Cardiomyocytes

CMs are terminally differentiated muscular cells that are connected end to end by gap junctions, allowing concerted contractile activity. Individual CMs constitute the basic units of gene regulation. It was established that CM gene expression underlies cellular phenotypes and determines cardiac function, but it remains elusive what gene programs regulate morphological remodeling and contribute to maintain or disrupt cardiac homeostasis.

The earliest single-cell-level sequencing studies of the adult heart started to emerge in 2017 [30,33,83,84]. A major challenge in profiling postnatal CMs is their unique size and shape, which limits their compatibility with many sequencing platforms. This was overcome by manual selection [28,85], large-particle FACS-sorting [29], and sequencing CM nuclei [9,13,33,86,87,88,89,90,91,92,93,94,95,96,97,98]. Single-nucleus sequencing has gained momentum as an alternative to single-cell sequencing, particularly with respect to profiling the large-size postnatal and adult CMs. A potential caveat with snRNA-seq of CMs is that adult CMs are often multinucleated, and, therefore, cell clustering and analysis based on single nuclei may be distorted by the proportion of multinucleated cells in the population, or by the properties of these distinctly nucleated cells. Fortunately, intact single CM sequencing addressed the latter issue, demonstrating that mono- and multinucleated cells seem to express similar sets of genes [33,60]. In a comprehensive side-by-side comparison of single-cell versus single-nucleus sequencing in differentiating hiPSCs, these two methods yielded similar results with respect to cell-type identification on days 0, 1, and 3 of cardiac differentiation. However, for both days 7 and 15, slight inconsistencies emerged. While both methods identified three transcriptionally similar clusters of cell types, the single-cell method uncovered an additional one enriched for the expression of *FLT1*, which was absent from the snRNA-seq data [42]. These observations suggest that scRNA-seq may be more sensitive at detecting cell subpopulations, possibly due to the greater abundance of transcripts in the cytosol.

Two large-scale sequencing studies of the adult human heart leveraging single-nucleus sequencing for CMs were published in recent years [9,13]. Tucker and co-workers [13] sequenced 287,269 nuclei from the four chambers of the normal human heart and identified nine major cell types, as well as over 20 cellular subtypes. They found the ventricular-specific expression of *HEY2* and *MYH7* and the atrial-specific expression of *NPPA* and *MYL4*. In addition to chamber specificity, CMs also exhibited sex-specific gene expression patterns. A total of 17 genes showed sex-based differential expression in CMs. Some of the genes were related to hormonal signaling, including *CRISPLD2* and *UGT2B4*, while others were associated with muscle contraction (*NEB* in men) and heart disease (*ZNF827* in female). Another large-scale study [9] revealed atrial and ventricular cellular types with different developmental origins and characteristics. In addition to the observation that ventricular myocardium was rich in *MYH7*, *MYL2*, *IRX5*, *IRX6*, *MASP1*, *HEY2*, and *PRDM16*, and atrial myocytes expressed *ALDH1A2*, *ROR2*, and *SYNPR*, they also identified five ventricular myocardial cell populations (vCM1–vCM5) and five groups of atrial CMs (aCM1–aCM5). Notably, both *MYH7* and *HEY2* were determined to be expressed in ventricular CMs in these two independent studies. Multiplex single-molecule fluorescence in situ hybridization (smFISH) was used to highlight the spatial arrangements and relationships of select cell populations. For example, vCM2, a subcluster highly expressing cardioprotective proteins *PRELID2*, *MYH6*, *CDH13*, and *HAMP,* was considerably enriched in more than 50% of right-atrium CMs versus 3% of left-atrium CMs. This study also identified gender-specific differences in CMs. Specifically, female hearts were associated with a higher proportion of ventricular CMs than male ones (56 ± 9% versus 47 ± 11%).

Our laboratory created the first cellular map of the adult human heart by sequencing intact CMs. We characterized a total of 11,492 single cells (including 3894 intact CMs) from different cardiac compartments, as well as from different health states (i.e., normal, heart failure of distinct etiologies, and recovery from heart failure) [8]. CMs in normal hearts exhibited apparent chamber-specific gene expression patterns. For example, left ventricular (LV) CMs were strongly enriched in functions related to oxidative phosphorylation, myocardial contraction, and circadian rhythms, marked by the rich expression of *SMYD1*, *ANKDR2*, and *FHL2*, while right ventricular (RV) CMs exhibited enrichment in signaling pathways, such as endoplasmic reticulum (ER) protein processing. Compared to ventricular CMs, left atrium (LA) CMs demonstrated greater involvement in cellular crosstalk (abundant expression of secreted and membrane proteins), as well as cardiac development, characterized by the expression of cardiac transcription factors *TBX5*, *SOX4*, and *MEF2A.* In addition to CM subtypes that correspond to specific chambers, we also identified a subtype that existed in both the ventricles and the atria (e.g., AV CMs) that surprisingly did not show any functional enrichments based on the analysis of differentially expressed genes. However, they highly expressed *SMARCA4*, a chromatin remodeler that maintains CMs in an embryonic state, which can also be induced upon stimuli, suggesting that AV CMs might represent a pool of less differentiated cells. CM heterogeneity was further complicated by age. Younger CMs expressed a large number of genes related to chemokine and cytokine signaling (e.g., *CCL4*, *DCN*, *CFD*, and *MGP*), while older CMs preferentially express genes involved in regulation of lipolysis and cGMP-PKG signaling pathways (e.g., *NPPA*, *IRS2*, and *AQP7*).

Collectively, CMs’ heterogeneity is most prominently determined by anatomic locations that can be traced back to distinct developmental origins and are further fortified by functional specifications, such as hemodynamics. The influence of gender and age on CM heterogeneity, while intriguing, is still under-investigated. Further understanding of gender-specific differences in CMs and other cardiac cell types may explain clinical observations and support epidemiological studies, while age-centric analyses may expand our knowledge of human cardiac maturation, aging, and regeneration.

CM heterogeneity may be further complicated by disease conditions, such as ischemia and hypertrophy [27]. In a mouse model of pressure overload-induced cardiac hypertrophy and subsequent failure, Smart-seq2-based scRNA-seq revealed seven subpopulations (C1–7) in CMs [28]. However, their relative proportions change drastically during disease progression. For example, a majority of sham-operated CMs (C6) were functionally enriched in GO terms pertinent to the mitochondrion and contraction fiber. On day 3 post-operation, functional enrichments of the predominant CM subcluster (C2) shifted toward translation, protein transport, and signal transduction, whereas 8 weeks following surgery, when hypertrophy progressed to heart failure, another subcluster, C7, which was implicated in cytoskeletal and actin binding deficiencies, rose to dominance [28]. Of note, the collection of CMs in this study was performed by manual pipetting. Although the throughput of 396 single CM transcriptomes was sufficient to support the study, 58–73 cells were collected for each time point, potentially missing important subtypes. In addition, hand-picking CMs could lead to bias due to manual selection of visually healthy cells.

As a more automated, unbiased, and higher-throughput approach, FACS with a large nozzle size (130 μm) was used to sort single adult CMs for sequencing. Among the four CM clusters detected from 426 healthy single cells, one was enriched in *Myoz2* expression, a protein potentially involved in the inhibition of cardiac hypertrophy. *Myoz2*-expressing CMs were localized on the surface of the epicardium. Notably, *Myoz2*-enriched CM cluster were found both in the embryonic heart and adult heart, suggesting the functional importance of this subtype throughout life [7,29]. A potential caveat in this study is the insufficient number of CMs captured, resulting in only 205 genes differentially expressed between healthy and injured CMs.

By contrast, Ren and co-workers profiled the transcriptomes of 11,492 single cells (including 5656 intact CMs) at different stages of pathological cardiac hypertrophy induced by pressure overload, using a nanowell-based approach. The CMs were partitioned into 10 distinct subpopulations, which were further grouped into four functional clusters based on transcriptome similarity. Unexpectedly, two of the functional clusters clearly expressed canonical endothelial (*Cdh5* and *Vwf*) or fibroblast markers *(Vim* and *Dcn*), which demonstrated elevated proportions during the middle stages of pathological remodeling, suggesting their involvement in adaptive responses of the heart. This finding highlighted the existence of atypical CM subtypes and their potential role in cardiac function [15].

### 3.2. Fibroblast

Cardiac fibroblasts are central actors in normal cardiac physiology and pathology. In adult hearts, fibroblasts are constantly modifying the microenvironment by depositing in the extracellular matrix (ECM). The heterogeneity of FBs seems less pronounced than CMs, with reports of only two or three major subsets in human [8,13] and mice [30] at the basal level. However, another study suggested additional subtypes, but still contained two major subsets that expressed canonical genes and displayed chamber specificity [9].

Under disease conditions, fibroblasts are activated and promote cardiac fibrosis, which is the abnormal accumulation of ECM in response to a pathological stimulus. Thus, the mechanistic basis of cardiac fibrosis has been sought after in an effort to find regulatable targets of this pathology. Myofibroblast-like subtypes with high expression of ECM proteins, such as *Postn*, were found to be induced in a mouse model of cardiac hypertrophy [15]. Interleukin 11 (IL-11), specifically expressed in fibroblast, was identified as the critical downstream effector of the pro-fibrotic factor transforming growth factor β1 (TGFβ1) [84]. *IL-11* was particularly highly expressed in fibroblast subclusters that showed features of TGFβ1 activation or ECM production. *IL-11* knockout attenuated cardiac fibrosis caused by either AngII infusion or transverse aortic constriction, demonstrating the critical role of FBs in response to profibrotic stimuli [84]. Expression of *TGFβ*-responsive genes was also identified in a human fibroblast subpopulation [9]. Cytoskeleton-associated protein 4 (CKAP4) was identified as a novel marker for activated fibroblasts in mouse hearts following ischemic injury. Its expression was validated in human ischemic hearts, and this finding correlated well with other established markers of activated fibroblasts (*POSTN*, *CTHRC1*, and *FN1*), indicating clinical relevance [29]. In human cardiac specimens of heart failure, transcription factor AEBP1 was abundantly expressed in activated fibroblasts and myofibroblasts, which co-expressed with fibrotic genes and ECM genes, indicating that AEBP1 may be a regulator of fibrosis in heart failure [16]. These studies demonstrate that, despite the use of scRNA-seq, current work regarding fibroblasts and cardiac fibrosis in disease is still focused on a single gene. It would be interesting to elucidate via trajectory analysis how fibroblasts become activated, and whether intervention with this subtype conversion could forestall fibrosis.

Fibroblasts are known to interact extensively with other cell types to maintain homeostasis or promote disease progression [89]. ScRNA-seq of non-CMs in normal adult mouse hearts revealed dense intercellular communication networks of cardiac cells, among which fibroblasts demonstrated the strongest communication capabilities. For example, FB can support macrophage function through the CSF1–CSF1R axis [90]. *Ngf* expressed by FBs could facilitate or maintain innervation of the heart through interaction with neurons [91]. In the same vein, FBs also expressed *Vegfa* and *Igf1*, which likely nourish neighboring endothelial cells [30]. Mature subtypes of FBs were also found to facilitate CM maturation through BMP signaling [61]. However, how fibroblasts interact with other cell types in cardiac diseases remains to be further characterized by scRNA-seq

### 3.3. Endothelial Cells

The endothelium is the innermost layer of cells lining the entire vascular system and is a sophisticated sensory and signal-processing center that controls virtually every cardiovascular function. Cardiac endothelial cells are a heterogeneous population that can reside in larger and small vessels in the myocardium, the lymphatics, and the endocardium. Cardiac abnormalities often target the endothelium, causing endothelial dysfunction [92]. Therefore, defining the functional diversity of ECs is essential to understanding human cardiac disease.

The heterogeneity of EC appears greater than that of FBs, with reported numbers of subpopulations between 4 and 10, depending on the specific study [8,9,13]. Capillary ECs, marked by the expression of *RGCC* and *CA429*, were found to comprise the majority (nearly 60%) of ECs in the heart. Other EC subpopulations included immune response-related subtypes [8,9], lymphatic subtypes [9,13], and artery- or venous-specific subtypes [8,9,13].

In human heart failure, activated ECs were observed to facilitate leukocyte recruitment through interaction with macrophages [16]. Intercellular communication between macrophages and ECs was also noted in atherosclerosis [31]. An *ACKR1^+^* subpopulation of ECs, characteristic of venous ECs [93], possessed the highest frequency of communication with CMs [8]. Importantly, this subtype was reduced in human heart failure. Injection of this population into infarcted mouse myocardium significantly attenuated cardiac function decline and fibrosis [8]. In another study using scRNA-seq to characterize the transcriptomic profiles of single cells in human cardiac arteries, one subset of coronary-artery-specific ECs was decreased in atherosclerosis, suggesting that it may protect against atherosclerosis and vascular calcification [31]. These findings underscore the importance of ECs and their crosstalk with other cell types in cardiac diseases.

### 3.4. Macrophages

Macrophages, while present at a much lower percentage in the myocardium compared to CMs, FBs, and ECs, play critical roles in homeostatic maintenance of the myocardium under normal conditions and in tissue repair after injury. In the steady-state heart, resident cardiac macrophages eliminate aging and dying cells and promote electrical conduction [94]. In the aging heart, the transition of the macrophage phenotype to the pro-inflammatory subtype leads to inflammation [95]. After myocardial infarction (MI), macrophages produce pro-inflammatory and anti-inflammatory mediators (cytokines, chemokines, matrix metalloproteinases, and growth factors), engulf dead cells, and promote angiogenesis and scar formation [95]. The biological functions of macrophages are closely related to individual subtypes and polarization states and would therefore benefit from single-cell profiling.

Dick et al. [26] identified four distinct macrophage clusters in the healthy mouse heart, including the *Timd4* (phosphatidylserine receptor *TIMD4*) cluster, *Mhc-II* (major histocompatibility complex class II) cluster, Isg (interferon-stimulated gene) cluster, and Ccr2 (chemokine receptor CCR2) cluster. Each subtype was associated with defined functions, strongly suggesting that a division of labor among macrophages exists in the heart even under steady-state conditions. Litvinukova and co-workers identified 3 *LYVE1*^+^ macrophage populations in the healthy human myocardium, including tissue-resident MPs, monocyte-derived MPs, and antigen-presenting MPs [9]. The latter two interacted with an FB subpopulation via the CD74–MIF axis, an interaction that may suppress cardiac fibrosis and tissue damage [9]. Interestingly, an atypical subpopulation of MPs was uncovered in the healthy mouse heart that exhibited hybrid expression of both MPs and FBs, the biological function of which is currently unknown [30]. This finding illustrated the unique capability of scRNA-seq in discovering previously unrecognized cellular phenotypes.

Notably, the heterogeneity of macrophages changed with age. A flow cytometric analysis revealed only a single subset in neonatal mice, but an additional two in adult mice [26]. Ischemic injury also induced significant changes in the composition of macrophages. Specifically, resident macrophages in the infarct area were markedly reduced after the infarction, and partially recovered via slow in situ proliferation. Macrophages recruited upon injury demonstrated remarkable plasticity by adopting distinct phenotypes, yet still fell short of compensating for the reduction in protection conferred by their resident counterparts.

A scRNA-seq analysis of leukocytes from infarcted and non-infarcted mouse hearts revealed that, after ischemic cell death, the uptake of cell debris by macrophages in the heart activated interferon regulatory factor 3 (*IRF3*) and type I interferons (*IFNs*), thus promoting the lethal response to myocardial infarction. Disruption of *IRF3*-dependent signaling reduced the cardiac expression of inflammatory cytokines and chemokines and improved cardiac function and survival [85]. Genes upregulated in ischemic-injury-induced macrophage clusters were enriched in processes related to the deposition of ECM, suggestive of cardiac fibrosis [29]. The role of macrophages in cardiac hypertrophy and heart failure has also been elucidated by scRNA-seq. Upon initiation of pressure overload-induced hypertrophy in mice (TAC model), the heart transitions from a compensatory to a decompensated state, the hallmark of which is the reduction in ejection fraction, indicating the onset of heart failure. Macrophages were found to undergo subtype switching during this transition. Specifically, compared to 2 weeks after TAC, macrophage clusters at 5 weeks post-TAC showed a pronounced expression of inflammatory factors, including Interleukin 1 beta (Il-1b) and C-C motif chemokine ligands (Ccl) 2, 6, 7, 8, 9, and 24. The expression of *Lgals3* (galectin-3), a marker of proinflammatory macrophages, was elevated substantially by 5 weeks. These activated MPs interacted with CMs through ligand–receptor pairs, particularly VEGFB-FLT1. Soluble FLT1 is known to contribute to cardiac remodeling and heart failure [96]. Therefore, the induction of proinflammatory macrophages was a key event during decompensation of the heart. Importantly, the inhibition of MP activation during 2–5 weeks post-TAC, but not earlier, significantly improved disease outcome [15]. Similarly, a subtype of tissue-resident macrophages (CXCL8^+^CCR2^+^HLA^-^DR^hi^) was shown to play a proinflammatory role in failing human hearts, suggesting the inhibition of CXCL8 as a potential strategy to suppress inflammation in the failing myocardium [16]. As can be seen, the study of macrophages has yielded several possible treatment strategies of cardiovascular diseases whose translational potentials remain to be further explored.

### 3.5. Smooth Muscle Cells (SMCs) and Pericytes (PCs)

SMCs and PCs are mural cells that support blood vessels, with the former situated around larger vessels and the latter surrounding capillaries. Both SMCs and PCs are abundantly found in the heart and play key roles in vascular tone and vascular integrity, as well as in angiogenesis [97]. Their importance in cardiovascular homeostasis and dysfunction is emerging due to their pleiotropic effects [98].

In a normal adult heart, PCs are identified by the expression of *ABCC9* and *KCNJ8* [99]. PCs were clustered into four distinct subpopulations based on their single-cell transcriptomes. A ventricle-enriched cluster, PC1, expressed genes involved in cellular adhesion and migration (e.g., *NCAM2*, *CD38*, and *CSPG4*), and PC2 was an atrium-specific PC subtype. Both PC3 and PC4 exhibited hybrid gene expression, marked by the co-expression of PC genes and EC genes and CM genes, respectively. An RNA velocity analysis indicated PC3 as a transitional cell state between PCs and ECs, as has been reported previously [100]. However, more work is needed to determine whether PC4 was a technical artifact or a genuine, yet unknown, subtype. Similar observations were made in another study of the normal human heart, in which one of the two identified pericyte subclusters was enriched for EC marker VWF. However, the possibility of cell or RNA contamination was not ruled out, and PCs were also found to display striking chamber specificity. Single-cell transcriptome profiling in adult mice uncovered *Ngf* and *Ntf3* expression in PCs, suggesting their potential involvement in the nervous innervation of the heart [30]. Nonetheless, it remains to be validated whether this non-canonical expression of neuronal factors in PCs was due to species specificity or cell contamination.

Compared to all of the abovementioned cell types, smooth-muscle cells did not show marked heterogeneity in healthy mice [30] or humans [13]. In the study by Litvinukova et al., *MYH11*-expressing vascular SMCs were divided into two populations. One displayed robust expression of typical SMC markers, including *CNN1, ACTA2* and *TAGLN*, indicating an arterial origin, while the other expressed stem cell marker *LGR6* and the proliferation-related gene *RGS5*, suggestive of venous origin [9].

### 3.6. Small Cell Populations

Neuronal cells make up the cardiac nervous system. In a study of 3961 cells marked by *NRXN1*, *NRXN3*, and *KCNMB4,* six neuronal cell (NC) subpopulations were defined [9]. The predominant subpopulation, NC1, exhibited typical neuronal gene signatures. Subclusters NC2–4 displayed co-expression of FB, CM, and EC markers, respectively. NC5 expressed G-protein-coupled receptor *LGR5*, a Wnt signaling and stem cell marker [101], which has been implicated in cardiomyocytes differentiation in the outflow tract. Other proteins related to neuronal development and diseases (e.g., *PPP2R2B*, *LSAMP*, and *LPL*) were also found in this subtype. The final subpopulation, NC6, expressed *MBP*, *PRX*, and *MPZ* and was reminiscent of Schwann cells [102] expressing genes that encode components of myelin [9]. Another study defined a subset of neuronal cells expressing neural cell adhesion proteins *NRXN1*, *NRXN3* and *NCAM2* that was located in all four compartments of the heart. Sodium voltage-gated channel alpha subunit 7 (*SCN7A*) was the only ion channel marker established for this subset. Speculations were made that this subcluster likely originated from the intrinsic cardiac autonomic neural network [13].

Epicardial adipose tissue is present in the healthy human heart, and at greater abundance under pathological conditions, such as obesity or cardiomyopathy. Epicardial adipocytes in the human adult heart were found to express genes involved in the regulation of lipid droplet size and stability (*CIDEC* and *PLIN5*), fatty acid transport (*ADIPOQ*), inactivation of thyrotropin-releasing hormones (*TRHDE*), cell growth and proliferation, (*IGF-1*) and, surprisingly, *CD96*, a marker associated with natural killer and T cells. Their data supported the idea of epicardial fat as an endocrine organ.

Adipocytes are also found in the myocardial tissue itself [9]. Cardiac adipocytes are marked by the expression of *GPAM, FASN, ADIPOQ*, and *LEP60* [9]. A single-cell analysis revealed three distinct subpopulations within these adipocytes. The first subset expressed canonical adipocyte genes related to the PPAR pathway and lipid metabolism. The second one showed enriched expression of ECM-related genes, possibly representing fibrogenic adipocytes. The third subtype could be a population of dysfunctional adipocytes, due to their expression of inflammatory genes and cytokines [103].

## 4. Modeling Human Cardiac Development and Disease with Pluripotent Stem-Cell Derived CMs

**Figure 2:**
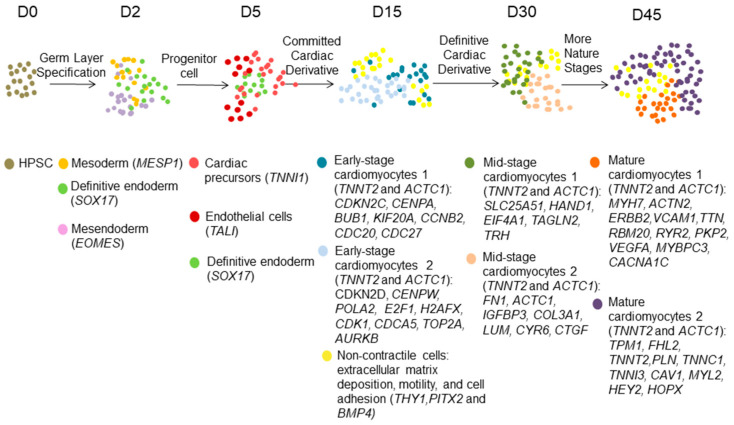
Cellular heterogeneity and gene expression during cardiac differentiation from hiPSC-CMs defined by single-cell transcriptomic profiling. On day 2, the differentiating population comprises cells corresponding to the mesoderm (*MESP1*), definitive endoderm (*SOX17*), and mesendoderm (*EOMES*). By day 5, cardiac precursors (*TNNI1*) and endothelial cells (*TALI*), and a persistent cluster of definitive endoderm are defined. On day 15 of cardiac differentiation, the population comprises 3 clusters, two expressing *TNNT2*, *ACTC1*, and cell cycle genes, representing early stage proliferative CMs; and a cluster of non-cardiomyocytes expressing genes enriched in processes, such as extracellular matrix deposition, motility, and cell adhesion (*THY1*, *PITX2*, and *BMP4*). The population on day 30 is composed of 2 clusters of mid-stage cardiomyocytes and a cluster of non-cardiomyocytes. By day 45, 2 mature CM clusters expressed sarcomeric (*MYH6*, *MYL2*, *TNNT2*, and *MYBPC3*) and calcium handling genes *(RYR2*, *PLN*). It is worth noting that the ventricular subsets marked by *MYL2* only appeared at the later stage of differentiation (D30–D90), in contrast to the atrial subsets (*MYL7*) that appeared earlier. This illustration is a schematic representation of scRNA-seq studies on hiPSC-CM differentiation by Churko et al. [40] and Friedman et al. [18].

Induced pluripotent stem cells (iPSC) allow for the in vitro development of CMs (hiPSC-CMs), which can closely mimic the genetic basis of cardiovascular diseases, and can also be used for drug discovery and toxicology testing, because they share transcriptomic, contractile and electrophysiological similarities with human CMs. Although iPSC-CMs seem to be a desirable cell model, much work is still being performed to promote maturation, enhance reprogramming and production efficiency, and control for heterogeneity. Therefore, many studies have used scRNA-seq to determine the signaling pathways involved in the transcriptional regulation of differentiation, identify cell types resulting from differentiation, and optimize and modify strategies for generating specific subtypes of cardiac and vascular cells (Table 3 and Figure 2).

### 4.1. Maturation

The maturity of in vitro derived CMs has long been a limitation in the use of these cells to model the human heart. Murine-ES-cell-derived CMs (mES-CMs) were shown to correspond E14.5 CMs of in vivo development, suggesting inadequate maturation of these in vitro derived cells [25]. More recently, a robust approach based on transcriptomic entropy, well-suited for cross-study comparisons, was applied to 45 scRNA-seq datasets and over 52,000 CMs to benchmark CM maturity. The entropy scores of human-induced PSC-CMs unveiled that these cells, even at relatively later time points, only corresponded to the start of the perinatal phase of in vivo CM maturation, suggesting that they cannot mature past the embryonic stage [104]. Nevertheless, tremendous efforts were undertaken to push these stem-cell-derived CMs toward maturation.

To understand the underlying mechanisms of insufficient maturation of hiPSC-CMs, Friedman et al. [18] applied scRNA-seq analysis to study gene regulation and fate selection that led to incomplete transcriptome activation. They employed an algorithm to construct the regulatory networks of hiPSC-CMs during differentiation. Comparing these with in vivo data unveiled *HOPX*, a protein expressed during CM specification at the progenitor stage, to be dysregulated in in vitro cardiac differentiation. Therefore, activation of HOPX may be a tool to promote in vitro maturation [18].

Glucose starvation (GS) is an established method to purify differentiating CMs [105]. Glucose has also been shown to suppress cardiac muscle maturation [106], suggesting that GS can simultaneously achieve the goals of purification and maturation. As a proof of concept, scRNA-seq of hiPSC-CMs at day 20 showed an elevated proportion of the late-stage CM subset, accompanied by a significant reduction in the proportion of non-CMs. It shows that GS treatment improves the purity and maturity of hiPSC-CMs. Several cardiac structural genes (*MYH6*, *MYH7*, *ACTN2*, and *TNNT2*) and contraction-related genes (*CACNA1C* and *RYR2*) were upregulated in the GS group, indicating better maturation [34].

The construction of co-cultures and 3D cultures is also a commonly used strategy to facilitate maturation of hiPSC-CMs [107]. In co-cultures of hiPSC-CMs and hiPSC-ECs, the maturity of the latter was improved by co-culture, whereas the maturation of hiPSC-CMs was not affected, or even reversed—as evidenced by the reduction in TCAP, a regulator of t-tubule structure and function—upregulation of pathways related to regulation of cell proliferation and cardiovascular system development [36]. In contrast, a comparison of bulk- and sc-RNA-seq data of tri-cellular (CM, EC and FB) microtissues with published datasets showed that in the integration of fibroblast into the 3D microtissue model led to more mature hiPSC-CMs, with increased expression of key cardiac sarcomeric genes: *TNNT2*, *MYL2*, *MYL3*, *MYL4*, *TNNI1*, *TNNI3*, *DES*, and *TCAP*. Metabolically, hiPSC-CMs in tri-cellular 3D microtissues also demonstrated favorable signatures, which are characterized by increased beta-oxidation-associated and tricarboxylic acid cycle (TCA)–associated genes, as well as concomitant decrease in glycolysis-related genes. This enhanced maturation was proposed to be the result of increased cAMP signaling and the assembly of *CX43* gap junctions [35].

### 4.2. Heterogeneity in Differentiation

Differences in CM differentiation protocols, time points analyzed, media composition, or purity of formed CMs may lead to different transcriptomes [108]. Modeling cardiovascular disease, screening for drug toxicity, and understanding cardiac development require stable and preferably homogeneous populations of CMs [109]. Therefore, understanding the cellular and molecular heterogeneity that emerges during differentiation is crucial for the application of iPSC-CMs [40,41,42].

A scRNA-seq of differentiating hiPSC-CMs captured at days 0 (pluripotency), 2 (mesoderm), 5 (progenitor), 15 (committed), and 30 (definitive) uncovered changing subpopulations in differentiating cells. Cells on day 2 comprised three subpopulations representing the three germ layers in embryonic development. Approximately one-third of cells at that stage showed significant enrichment for cardiogenic gene networks. Cardiac precursor, definitive endodermal, and endothelial cells were identified as the three subclusters at day 5. On the 15th and 30th days, a subset of CMs and another subset of non-CMs were present in the general population [40]. Likewise, the commercially available iCell CMs were also divided into two subclusters [41]. Curiously, the minor subpopulation (10.8%) had clear signatures of cell-cycle gene expression, but lacked enrichment of the expression of other cell type or pluripotency markers, ruling them out as non-CMs or progenitor-like CMs [41]. It is worth noting, however, that the choice of the specific sequencing method may directly influence data accuracy and representativeness [41]. Direct comparison between single-cell and single-nucleus RNA-seq of differentiating hiPSC-CMs on day 15 detected four and three subclusters, respectively, indicating that sequencing whole cells may be more sensitive in subtype identification [42]. Other protocols have generated hiPSC-CMs with more subpopulations. For example, hiPSC-CMs at day 20 of differentiation could be divided into five subpopulations, including late-stage CMs, middle-stage CMs, CMs with lower expression of cardiac markers, ectoderm cells, and endothelial cells [34]. In another study, hiPSC-CMs, at day 30 of differentiation, were shown to consist of six subpopulations [40]. An scRNA-seq of a total of 10,376 cells from days 0, 5, 14, and 45 of differentiation of hiPSC-CMs was performed to characterize the source of hiPSC-CM heterogeneity [40]. A total of 20 distinct clusters were observed from all time points, indicating dramatic heterogeneity during cardiac differentiation. Additional bulk RNA-seq with cells spread across 13 time points was applied to dissect time-dependent changes in the transcriptome. Not surprisingly, the composition of cells changed with time, with most changes occurring before day 30. Specific markers emerged during the time course: the expression of *NR2F2* and *ISL1* was first observed at the early time points of differentiation (days 3 to 4), TBX5 was expressed at the intermediate time points (days 5–14), while *HEY2* and *HOPX* were expressed at the late stage of differentiation (day 9, 14, 30, and 90). In addition, manipulating the expression of these specific regulators made it possible to modify the cellular composition of the hiPSC-CMs [40].

Whether transcriptional subtypes correlate with functional subtypes has been a long-standing question in the field. Parallel analysis of electrophysiology by a genetically encoded voltage indicator, ArcLight, and gene expression by scRNA-seq showed that CM subtype classifications based on these respective parameters did not align. Specifically, at the functional level, both day 12 and day 40 could be clearly classified into ventricular or atrial subtypes, whereas there were not such distinctions transcriptionally [43].

### 4.3. Patient-Specific hiPSC-CMs

One of the most prominent applications of hiPSC-CMs is to implement personalized medicine and model genetic diseases, including congenital heart diseases [108,110]. The application of scRNA-seq can therefore be used to discover cellular phenotypes, dissect underlying mechanisms, and predict intervention targets [46,47,48,49].

Hypoplastic left heart syndrome (HLHS) is the most common manifestation in the spectrum of left ventricular outflow tract obstruction defects associated with ventricular hypoplasia. ScRNA-seq was used to investigate the consequences of cell subtype formation in HLHS cells, using iPSC-CM differentiation. Ten clusters of CMs of a spectrum of maturation statuses were recovered. HLHS cells were almost exclusively contributed to the cluster of early cardiac progenitors and were virtually absent from the subset of more mature CMs, suggesting severe maturation defect [46]. Another scRNA-seq study of hiPSC-CMs (day 30) from control and HLHS patients showed that, while both groups displayed appropriate ventricular differentiation, mitotic cells within the general population distinguished HLHS from control. Specifically, HLHS cells had significantly lower levels of genes related to mitochondrial function and metabolism, and this was the underlying reason for the observed impairment in contractility [48].

Hypoplastic right heart syndrome (HRHS) is another type of cardiac malformation that is more commonly seen in the Asian population, and it is the result of the underdevelopment of the right ventricle and pulmonary or tricuspid valvar atresia. Moreover, hiPSC-CMs cells derived from pulmonary atresia with intact ventricular septum (PAIVS) patients also exhibited reduced expression of genes associated with heart contraction and maturation. Thus, in contrast to HLHS, the functional deficit in PAIVS was primarily due to impaired contractility [47].

Aside from congenital heart diseases, other genetic cardiac disorders have also been studied via scRNA-seq. For example, Lamin A/C gene (LMNA) gene mutations, which are known to cause a variety of heart diseases, were studied by scRNA-seq of iPSC-CM lines of patients with LMNA (c.357-2A > G) mutations [49]. Compared to control cells, patient iPSC-CMs exhibited lower expression levels of several cytoskeletal (e.g., *TMOD1*, *SGCA*, and *DMD*) and ion channel/contractility (e.g., *RYR2* and *SCN5A*) genes, which correlated with force generation on the ‘heart-on-a-chip’ platform.

Collectively, accumulating efforts are aim at using hiPSC-CMs to model the human heart and pertinent diseases. The use of scRNA-seq has successfully illuminated the heterogeneity in these differentiating cells, and has aided in the dissection of disease mechanisms. Additional future work on the single-cell pharmacological or toxicological responses [111] of hiPSC-CMs will expand their utility.

### 4.4. Exploring Developmental Processes

CMs derived from pluripotent stem cells can be a useful tool to mimic and understand the spatiotemporal molecular underpinnings of cardiac development. The combined use of scRNA-seq, ATAC-seq, and ChIP-seq was applied to decipher the epigenetic control of cardiac and neuronal differentiation from hESCs. ARID1A, a subunit of the SWI/SNF chromatin remodeling complex, was essential for the opening of chromatin regions for the promoters of cardiogenic genes, whose deletion significantly impaired cardiac differentiation from hESCs [44]. The scRNA-seq of the hESC-to-CM differentiation process at six key time points (days 0, 2, 5, 9, 14, and 60) uncovered crosstalk between CPCs and the endodermal lineage to be involved in cardiac lineage commitment. This intercellular communication led to increased ETS Proto-Oncogene 1 (*ETS1*) expression in cardiac progenitors. The *ETS1* occupancy was substantially increased at cardiac genes at day 9 of differentiation, the time point of cardiac lineage specification. These observations underscore the importance of cell–cell interaction in cardiac lineage commitment, and the potential advantage of cellular heterogeneity during in vitro cardiac differentiation [45]. However, whether the above findings also apply to in vivo human heart development remains to be further explored.

### 4.5. Improve Direct Reprogramming Efficiency

Direct reprogramming of cardiac fibroblast into CMs is an exciting strategy to replenish lost myocardium. However, the reprogramming process is asynchronous by nature and produces a heterogeneous population of cells on route to induced CMs (iCMs). Therefore, understanding the continuum of cellular changes and improving reprogramming efficiency has become an important area of research.

Because iCMs appear as early as day 3 of reprogramming, scRNA-seq was used to profile the cell population at this stage. As expected, cells formed a continuum of four subpopulations based on differential expression of fibroblast and CM genes. Splicing factor *Ptbp1* was recognized as a critical barrier to the acquisition of CM-specific splicing patterns in fibroblasts, whose depletion was sufficient and led to improved iCM reprogramming efficiency [38]. While the results obtained in mice are enticing, the ultimate goal of reprogramming is to perform it in human cells, which can be more complex. To study the cellular and molecular dynamics of cell fate conversion during human iCM induction, Zhou et al. [37] performed scRNA-seq on hiCM at multiple time points during reprogramming (D3, 5, 7, and 9). MiR-133 was shown to inhibit the proliferation of human cardiac fibroblasts (hCF), while loss of the latter during in vitro reprogramming was a prerequisite for the transition of fate. The silencing of several miR-133 targets improved iCM reprogramming. The authors also discovered that immunity is critical to the fate of CMs during hiCM reprogramming by affecting the DNA methylation status of heart loci. Eventually, two routes of cell fate conversions were identified: a reprogramming and a refractory route, through which cells turn back to their starting point [37]. It would be interesting to investigate whether manipulation of the refractory could result in enhanced reprogramming efficiency. The same team further employed an integrative analysis of scRNA-seq and scATAC-seq data to unveil changes in the epigenetic landscapes during the early phases of transdifferentiation. Transcription factor Smad3 was found to play opposing roles during reprogramming, as it was inhibitory at the initiation stage (D0–3), but promotive during the middle stages (D3–12) In addition, silencing of cardiac fibroblast transcription factor Tcf21 and AP-1 subunit Fos dramatically boosted reprogramming efficiency [39].

## 5. Perspectives and Significance

ScRNA-seq, as well as related techniques, have already produced 442 human cell atlases since its launch in 2012. Numerous other resources and research studies in other model systems were also fueled by these technologies. Among them are three maps of the human adult heart [8,9,13] and two of the developing human heart [7,14], offering unprecedented resolution of the biodiversity and heterogeneity of cells, of cell fate conversions and lineage commitment, of cellular crosstalk, and of subcellular events and mechanisms that underlie cellular phenotypes, findings that would not have been possible without single-cell techniques. Compared to the large body of single-cell work dedicated to deciphering cardiogenesis, embryonic cardiac development, and adult cardiac homeostasis and dysfunction, few studies were directed at postnatal development and maturation [60]. Massively parallel snRNA-seq of postnatal day (P) 6 and P10 mouse hearts by sNucDrop-seq [112] showed a decreased proportion of proliferating CMs and a concomitant increase in the percentage of mature CMs, indicating active differentiation and maturation of CMs within this time window [60]. The factors that drive CM maturation, though, remain an incompletely answered question.

On the other hand, the isolation of single cells destroys information on their spatial localization within the tissue, as well as intercellular information transfer. The heart is the first fully functionalized physical organ during human embryonic development. Its complex development processes, such as the differentiation of the cardiac tube from the mesoderm, the bending of the heart tube to form four cavities, and the septation of the outflow tract into the trunks of aorta and pulmonary artery, have all been well portrayed at the general level. Although scRNA-seq has already added a fair amount of new knowledge to our previous understanding of heart physiology and disease, many of the other aspects of molecular changes, and how they relate to each other, have not yet been fully resolved. Therefore, integrative analysis of single-cell transcriptomes and other omics, particularly in the form of scMulti-omics, is an actively developing field of study, and is expected to transform our understanding of biology. However, the application of scMulti-omics is still in its early stages. Many technical and computational limitations need to be overcome in order to improve data quality, fidelity, and insights gained from scMulti-omics analyses. We anticipate these technologies be used to accelerate the discovery of cell biomarkers for monitoring endpoints in clinical trials, to predict responses to treatment, and to provide analytical methods and automated platforms for pharmaceutical applications.

## Figures and Tables

**Table 1:** Summary of studies of heart development.

Reference	Species	Developmental Stage	Technique (Number of Cells/Nuclei)	Target Tissues/Cells
Chan et al. [59]	Mouse	Mouse ESC-derived embryoid bodies day 4	scRNA-seq (42 cells)	Dissociated cells from embryoid bodies
Lescroart et al. [20]	Mouse	Embryonic day 6.25 and 7.5	scRNA-seq (513 cells)	*Mesp1*^+^ or *Mesp1* KO CPCs
Tyser et al. [21]	Mouse	Embryonic day 7.75–8.25	scRNA-seq (3105 cells) Multiplexed RNA imaging	Manual microdissection to isolate the anterior cardiac region
Jia et al. [22]	Mouse	Embryonic day 7.5, 8.5, and 9.5	scRNA-seq and scATAC-seq (>1258 cells)	*Isl1*^+^ or *Nkx2.5*^+^ CPCs
Li et al. [12]	Mouse	Embryonic day 8.5, 9.5, and 10.5	scRNA-seq (2233 cells)	Microdissected embryonic heart tissues of each chamber
Xiong et al. [23]	Mouse	Embryonic day 7.75, 8.25, and 9.75	scRNA-seq (1231 cells), CHIP-seq	*Isl1*^+^ or *Nkx2.5*^+^ CPCs
de Soysa et al. [17]	Mouse	Embryonic day 7.75, 8.25, and 9.25	scRNA-seq (36,777 cells)	CPCs from control and *Hand2*-null embryos
DeLaughter et al. [25]	Mouse	Embryonic day 9.5, 11.5, 14.5, and 18.5; postnatal day 0, 3, and 21	scRNA-seq (1133 cells)	Microdissected embryonic heart tissues of each chamber
Cui et al. [14]	Human	5–25 weeks gestation	scRNA-seq (3842 cells)	Anatomically informed cardiac cells from human embryos
Goodyer et al. [24]	Mouse	Embryonic day 16.5	scRNA-seq (22,462 cells)	Cells from three zones of microdissected hearts: sinoatrial node region, atrioventricular node/His region, and bundle branch/Purkinje fiber region
Asp et al. [7]	Human	4.5–5, 6.5, and 9 weeks gestation	scRNA-seq (3717 cells), spatial barcoding, and in situ sequencing	Human embryonic and fetal cardiac cells
Hu et al. [60]	Mouse	postnatal immature heart	snRNA-seq (15,082 cells)	isolated nuclei from postnatal hearts (P6, P10)
Wang et al. [61]	Mouse	Postnatal day 1, 4, 7, 14 and 56	scRNA-seq (2137 cells)	CMs and non-CMs from left ventricles

**Table 2:** Summary of studies of adult heart.

Reference	Species	Sample Category	Technique (Number of Cells/Nuclei)	Target Tissues/Cells
Dick et al. [26]	Mouse	adult heart	scRNA-seq (8283 cells)	Macrophages and dendritic cells from adult heart, cardiac mononuclear cells from adult heart (non-operated or D11 post-MI)
Tucker et al. [13]	Human	adult heart	snRNA-seq (287,269 cells)	Tissue samples taken from the lateral aspect of the four cardiac chambers from potential transplant donors
Litvinukova et al. [9]	Human	adult heart	scRNA-seq (123,893 cells), snRNA-seq (363,213 nuclei), and multiplexed RNA imaging	Full-thickness myocardial biopsies from the left and right atria, left and right ventricles, and interventricular septum and apex from deceased transplant organ donors
Wang et al. [8]	Human	adult heart	scRNA-seq (21,422 cells)	CMs and non-CMs from biopsy samples of LA and LVs of normal, failed, and recovered adult human hearts
Yekelchyk et al. [27]	Mouse	adult heart	scRNA-seq (>586 cells)	CMs from both healthy and hypertrophic ventricles
Nomura et al. [28]	Mouse and human	adult heart	scRNA-seq (396 cells)	CMs isolated from LVs of mice after sham surgery or 3 days and 1, 2, 4, and 8 weeks after TAC/DCM patients or normal control
Gladka et al. [29]	Mouse	adult heart	scRNA-seq (426 cells)	Cells from the infarct and border zone region from infarcted heart at day 3 post-MI or sham
Ren et al. [15]	Mouse and human	adult heart	scRNA-seq (11,492 cells)	CMs and non-CMs isolated from LVs of mice after sham or 2, 5, 8, and 11 weeks after TAC/end-stage DCM, HCM patients, and control
Skelly et al. [30]	Mouse	adult heart	scRNA-seq (10,519 cells)	Non-CMs from the heart
Rao et al. [16]	Human	adult heart	scRNA-seq (200,615 cells)	Non-CMs from left and right ventricle of DCM hearts and infarcted and non-infarcted area of ICM hearts
Hu et al. [31]	Human	adult heart	scRNA-seq (>100,000 cells)	Cells from human aorta, pulmonary artery, and coronary arteries collected from patients undergoing heart transplantation
King et al. [83]	Mouse	adult heart	scRNA-seq (4215 cells)	Leukocytes isolated from wild-type and *Irf3*-null heart at day 4 post-MI or sham
Wang et al. [32]	Mouse	adult heart	scRNA-seq (12,779 cells), scATAC-seq (9524 nuclei)	Heart non-myocytes
See et al. [33]	Mouse and human	adult heart	snRNA-seq (359 nuclei)	Nuclei of CMs isolated from LVs of mice 8 weeks after TAC or sham surgery/end-stage DCM patients or control

**Table 3:** Summary of studies of induced pluripotent stem cells.

Reference	Species	Sample Category	Technique (Number of Cells/Nuclei)	Target Tissues/Cells
Friedman et al. [18]	Human	hESC and hiPSC and derivative	scRNA-seq (43,168 cells)	hESC- and hiPSC-derived cells (D0, D2, D5, D15, and D30)
Ni et al. [34]	Human	hiPSC and derivative	scRNA-seq (13,827 cells)	hiPSC-derived cells (D20)
Giacomelli et al. [35]	Human	hESC and hiPSC and derivative	scRNA-seq (16,307 cells)	3D and 2D cultured hiPSC-derived cells
Helle et al. [36]	Human	hiPSC and derivative	scRNA-seq (4000 cells)	co-cultured hiPS-CMs and hiPS-Ecs (48 h)
Zhou et al. [37]	Human	hiCM	scRNA-sEq (652 cells)	cardiac fibroblast reprogramming into CMs (D0, D3, D5, D7, and D9)
Liu et al. [38]	Mouse	iCM	scRNA-seq (454 cells)	cardiac fibroblast reprogramming into CMs (D3)
Wang et al. [39]	Mouse	iCM	scATAC-seq (19,397 nuclei)	cardiac fibroblast reprogramming into CMs (D3)
Churko et al. [40]	Human	hiPSC and derivative	scRNA-seq (10,419 cells)	hiPSC-derived cells(D1, D5, D14, D30, and D45)
Schmid et al. [41]	Human	iCell	scRNA-seq (1421 cells)	Commercially available iCell cardiomyocyte (Fuijifilm Cellular Dynamics)
Selewa et al. [42]	Human	hiPSC and derivative	scRNA-seq (25,475 cells), snRNA-seq (22,025 nuclei)	hiPSC-derived cells or isolated nulcei (D0, D1, D3, D7, and D15), nuclei from adult heart tissue (68Y)
Biendarra-Tiegs et al. [43]	Human	hiPSC and derivative	scRNA-seq (85 cells)	hiPSC-CMs (D12 and D40)
Liu et al. [44]		hESC	scRNA-seq (20,455 cells), ATAC-seq and ChIP-seq	WT and ARID1A^−/−^ hESCs
Ruan et al. [45]	Human	hESC and derivative	scRNA-seq (6879 cells)	hESC- derived cells (D0, D2, D5, D9, D14, and D60)
Krane et al. [46]	Human	iPSC-CMs	scRNA-seq (10,870 cells)	iPSC-CMs from patients with HLHS and control (D14)
Lam et al. [47]	Human	iPSC-CMs	scRNA-seq (25,059 cells)	Time-matched D30 hiPSC-CMs, D10 hCAS, and D10 hCTS from two healthy subject and two PAIVS hiPSC lines
Paige et al. [48]	Human	iPSC-CMs	scRNA-seq (9899 cells)	iPSC-CMs from one control and one HLHS patient (D30)
Mehrabi et al. [49]	Human	iPSC-CMs	scRNA-seq (25,619 cells)	iPSC-CMs from 2 control and 2 LMNA patients with a (c.357-2A > G)
Paik et al. [50]	Human	iPSC-ECs and derivative	scRNA-seq (5673 cells)	iPSC-ECs differentiation (D8 and D12)
McCracken et al. [51]	Human	hESC-ECs and derivative	scRNA-seq (105,727 cells)	hESC-ECs differentiation (D8)
